# A Review of Nitrogen Use Efficiency of Dairy Replacement Heifers: Improving Management Practices and Minimizing Nitrogen Losses

**DOI:** 10.3390/ani15071031

**Published:** 2025-04-03

**Authors:** Antigoni Plomaritou, Mikenzie Hanlon, Dimitrios Kantas, Konstantinos Georgakoudis, Eleni Dovolou, Andreas Foskolos

**Affiliations:** Department of Animal Science, University of Thessaly, Campus Gaiopolis, 41500 Larisa, Greece; anplomaritou@uth.gr (A.P.); mikenziehanlon@gmail.com (M.H.); dkantas@uth.gr (D.K.); kostas.georgakoudis@gmail.com (K.G.); entovolou@uth.gr (E.D.)

**Keywords:** nitrogen, efficiency, dairy heifers, nutrition, growth, management

## Abstract

Within the agricultural sector, dairy cattle farming systems are of the main ammonia emitters contributing to nitrogen (N) pollution. As a main strategy to mitigate N pollution, increased N use efficiency (NUE) of dairy cattle farms has been proposed. Dairy replacement heifers are an important aspect of dairy herds, and improving their NUE is a step toward addressing the issue holistically. The aim of the current review was to describe and assess heifer nutritional practices that affect NUE and examine protein supplementation to minimize N excretion in the environment. Heifer management practices related to growth and nutrition appear to have an important role on improving overall farm NUE. In particular, age at first calving, dietary crude protein level and rumen degradable protein level appear to be among the factors that affect N losses during the growth period. From a management point of view, frequent body weight monitoring at growth benchmarks and the use of mechanistic nutritional models are highly desirable to increase NUE under on-farm conditions.

## Simple Summary

Dairy cattle farming has been criticized for its contribution toward environmental pollution. Among various pollutants deriving from dairy herds, ammonia emissions are the main concern, as they contribute to air quality degradation and nitrogen pollution. The improvement of nitrogen use efficiency of dairy systems has been proposed as an important mitigation strategy. Dairy replacement heifers are usually overlooked when it comes to implementation of mitigation strategies. However, they are a vital part of dairy herds. Heifers’ growth and nutritional practices appear to affect the nitrogen losses of a dairy farm. Therefore, proper rearing management of replacement heifers plays a crucial role in the overall improvement of farm nitrogen use efficiency.

## 1. Introduction

Under natural conditions, non-reactive atmospheric di-nitrogen (N_2_) is converted to reactive N (N_r_) through biological fixation, burning of biomass or lightning [1], while N_r_ immobilization is achieved through the denitrification process [2]. However, the availability of N_r_ is limited, and humanity, based on the Haber–Bosch process, managed to produce NH_3_ and develop synthetic fertilizers to increase crop productivity [2]. Agriculture, fossil fuel combustion and industrial production were highly dependent on N_2_ fixation to produce various forms of N_r_, which include inorganic forms such as NH_3_, ammonium (NH_4_^+^), nitrates (NO_3_^−^), nitrogen oxides (NO_x_) and nitrous oxide (N_2_O) and organic compounds such as urea, amines and proteins [3,4,5]. Initially, the use of N_r_ benefited society and industry, contributing to a population increase and life quality improvement [1]. In a balanced system, N_r_, through the form of manure, will be deposited as natural fertilizer on fields, and eventually N_r_ will be used by plants for their growth requirements [2]. However, the extended use of N_r_ through synthetic fertilizer application and combustion for energy supply and transportation purposes caused additional unintended N_r_ flows, disrupting the natural denitrification back to N_2_ [2,3]. More recently, the accumulation and creation of N_r_ in systems is higher than the rate of denitrification [3,5]. These N_r_ tradeoffs among terrestrial, aquatic and atmospheric ecosystems have caused multiple environmental effects (soil and water acidification, eutrophication and contribution to GHG emissions), a phenomenon known as the N cascade. This concept describes the mobility of N_r_ molecules through ecosystems, while the excessive release of N_r_ compounds is generally referred to as N pollution [2].

Dairy cattle farming has been linked with N pollution, as agricultural and manure management practices of the past resulted in the deposition of excessive Ν amounts into the ecosystem [4,6]. Moreover, nutritional practices, such as feeding protein above an animal’s requirements, contributed to the problem [7]. Νon-utilized N in milk or meat is excreted through urine and feces into the environment and enters into an already N-saturated ecosystem [8]. Various pollutants are generated from manure deposition such as N_2_O, a potent greenhouse gas, NH_3_ and NO_3_^−^, whose leaching contributes to soil and water pollution [9]. Ammonia emissions are a major concern as, among other issues, they consist of the precursor for particulate matter formation, with a negative impact on human health [10]. Furthermore, agriculture and livestock farming appear to contribute the most to NH_3_ emissions. Malherbeet al. [11] estimated NH_3_ emissions within the European Union (EU) from different sectors. They reported that agriculture is the main sector emitting NH_3_ in the air and accounts for 94% of the total NH_3_ emissions, while the livestock sector accounts for 72% of agricultural NH_3_ emissions [11]. Moreover, among different types of livestock, cattle contribute the most to NH_3_ emissions, accounting for 51.3% of the total NH_3_ emissions in the EU, followed by swine at 27.5% [11].

The increase in N use efficiency (NUE) of dairy farming systems has been proposed as an initiative to mitigate nitrogen pollution [2]. The NUE is a fraction of the N input into the system that turns into N output, and at an animal level, it can be defined in terms of g N in product per g N consumed [12]. Identifying all parameters that influence NUE at an animal and farm level may further improve N utilization and minimize N losses to the environment [12]. Most efforts in mitigating N pollution focus on the lactating herd, with heifers receiving less attention. However, replacement heifers are a major part of the dairy herd, as their age at first calving (AFC) and N losses during the growth period negatively impact overall farm NUE [13,14].

Therefore, the aim of the current review was to describe heifer rearing practices that affect the NUE of replacement heifers and explore management factors that may improve NUE and minimize N losses in the environment.

## 2. Nitrogen Pollution of the Dairy Herd and the Role of Dairy Replacement Heifers

Understanding the mechanism of NH_3_ volatilization and, consequently, N deposition into the ecosystem is a crucial step toward the development and evaluation of N pollution mitigation strategies. The formation and volatilization of NH_3_ starts instantly with urine and feces excretion [15]. The urea in urine, which represents 50–90% of total urinary N [16], is rapidly hydrolyzed by the enzyme urease that is produced by rumen microbes and excreted in feces [15]. As a result, NH_3_ and NH_4_^+^ are formed [17], with 4–41% of the N present in urine being volatilized, while only 1–13% of the N present in feces is volatilized and lost [16]. The volatilization rate is highly dependent on the NH_3_ concentration in the manure, with the main contributor being urinary N, while the N contained in feces is mainly organically bound and less volatile [10,15]. In total, the loss of N in the form of NH_3_ accounts for 25–50% of manure N excretion [15]. Regarding dairy cows, NH_3_ emissions are well studied. Hristov et al. [15], in a meta-analysis, reported NH_3_ emissions of 59.0 g/d per cow, ranging from 0.8 to 250.0 g/d. A metanalysis conducted by Bougouin et al. [18] reported average NH_3_ emissions of 60.1 g/d per cow, ranging from 7.8 to 213.0 g/d per cow. However, regarding replacement heifers, NH_3_ emissions data are significantly less available. Powell et al. [19], using 16 heifers at the age of 17 months old which were allocated into four chambers for testing four different types of bedding, reported NH_3_ emissions from the dairy heifers of 12.3, 24.7 and 25.5 g/d for winter, summer and fall, respectively. Lascano et al. [20] conducted two experiments of 24 dairy heifers each, testing different forage-to-concentrate ratios, and measured NH_3_ emissions from the manure of a heifer’s pen floor with the use of a portable flux chamber. It was reported that for heifers which were 9 and 14 months old, NH_3_ emissions ranged from 9.4 to 12.2 g/d and 19.0 to 24.3 g/d, respectively [20]. Even though the age range was wide, available data in the literature regarding actual NH_3_ emissions derived from heifers were limited. Thus, for the purpose of the current literature review, based on the work of Powell et al. [19] and Lascano et al. [20], it can be calculated that, on average, dairy heifers emit 17.3 g of NH_3_ per day.

The findings concerning both heifers and cows indicate that there is much variation regarding NH_3_ emission estimates. Multiple factors result in this variation, relating to either the animal, such as its stage of growth and body weight, or environmental factors, such as the housing and bedding type, manure handling system, ventilation, season and environmental temperature [21]. Bougouin et al. [18] conducted a meta-analysis and found that in dairy cows, on average, 9.3% of the N intake was lost as NH_3_ (range: 1.6–30.0%), while NH_3_ emissions were moderately affected by N consumption and the crude protein (CP) level (R^2^ = 0.31). Furthermore, in the mixed-effects model, they included the milk yield, dry matter intake (DMI) and CP level and explained 45.5% of the heterogeneity, indicating positive effects from the CP content and DMI on NH_3_ emissions [18]. Similarly, concerning dairy heifers, Marini and Van Amburgh [22] observed that when increasing the N level of provided diets, an increase in urinary N excretion was observed without affecting fecal N excretion. Hence, a potential decrease in N losses through NH_3_ volatilization can be realized by decreasing the provided CP of the diets.

As NUE is the system’s N output divided by the N input into the system [23], an increase is feasible either through an increase in the numerator or a decrease in the denominator. At a dairy cow level, the measurement of NUE is the conversion of dietary N into milk N, and it is defined as milk N use efficiency (MNE), expressed as a percent ((g of milk N content/g of dietary N) × 100) [12]. In dairy cows, N utilization is relatively low, with MNE presenting high variation and ranging from 14.0 to 45.3% [24]. An increase in MNE can be achieved through the adaptation of N intake at lower levels or an increase in the N contained in the milk. The milk protein content is positively correlated with the milk yield [18]. Thus, an increase in milk production may further improve MNE, provided that N intake does not increase [24]. Similarly, in replacement heifers, increasing N retention or reducing N intake without affecting growth might improve NUE at the animal level. Indeed, several studies investigated the CP level, N retention and growth and observed that lower-CP diets reduced N excretion without compromising growth [25,26,27].

At the farm level, MNE appears to be among the factors that highly impact the lifetime nitrogen use efficiency (LNE) of a farm [13]. Aside from MNE, a farm’s LNE is affected by the calving interval, a reproduction parameter, and AFC. A negative correlation was observed between the AFC, LNE and replacement NUE [13]. Typically, heifers enter the lactating herd at an AFC of 24 months old. The extension of the non-productive period, which results in higher AFC, highly affects the total N requirements for heifer growth, meaning that higher N losses can occur, leading to reduced farm LNE [13]. The necessary number of heifers with different AFC targets can be estimated with the following equation: heifers required = herd size × (AFC/24) × replacement rate × (1 + mortality rate). Thus, for a 100-cow dairy farm with a 34% replacement rate, 6.4% mortality rate [28] and AFC targets of 22, 24 or 26 months, the necessary number of heifers required annually is 33.2, 36.2 or 39.2, respectively. Based on available studies on heifer NH_3_ emissions, it was calculated that 17.3 g/d of NH_3_ is emitted per heifer [19,20]. Thus, NH_3_ emissions at the herd level are estimated to be 209.6, 228.6 and 247.5 kg/year when the AFC is 22, 24 and 26 months, respectively. It should be mentioned that the intensity under which heifers grow after weaning directly affects the onset of the breeding process and ultimately the AFC [29,30,31]. Thus, controlling the AFC is a crucial first step toward reducing N losses.

Replacement heifers comprise a major part of the dairy herd and contribute significantly to the total farm N excretion. It is evident that regarding heifers, N losses are affected by multiple factors that are relative to growth, nutrition and reproduction parameters, highlighting the importance of addressing the issue holistically. Overall, improving N utilization, meaning the N consumption of a heifer and its retention in the heifer’s body, combined with shorter rearing periods, can potentially further minimize N losses. Hence, better heifer growth and conception rates may improve AFC, shortening the non-productive period. Moreover, proper nutrition and reproduction management of heifers enable programming the first calving and might serve as a tool to both mitigate environmental impact and improve overall farm NUE.

## 3. Growth Targets of Dairy Heifers

The extension of the rearing period is highly affected by the age at which heifers are bred, while the onset of the breeding process depends on growth and nutritional parameters [32]. Setting ADG targets and formulating diets to achieve them can be an effective strategy to control the age of breeding and therefore achieve the desired AFC.

Currently, one of the main rearing goals of replacement heifers is to attain an AFC at 22–24 months [33,34,35]. Toward this aim, different growth benchmarks should be met throughout the rearing process. Growth targets for heifers are expressed as a percentage of the mature body weight (MBW). Notably, lactating cows reach mature size at third parity. Hence, the MBW is estimated as the average BW of cows in their third lactation within the herd [32,36]. However, body size is a heritable trait [37], and within a herd, the selection of animals may be toward animals with large or small frame sizes [36]. Thus, growth targets are breed- and herd-specific [38].

Coefficients based on the MBW have been suggested to facilitate the determination of the required BW at various growth benchmarks. Van Amburgh et al. [39] suggested at least 82% of the MBW as a BW target for first calving. Fox et al. [40], based on a previously developed model [41] and data adapted from Van Amburgh et al. [39], proposed BW postpartum at 85% of the MBW and 92% and 96% of the MBW as target weights for second and third parity, respectively. The National Research Council (NRC; 2001 [42]) further modified the recommendations of Fox et al. [40] and suggested BWs of 82, 92 and 100% of the MBW for first, second and third calving, respectively. Hoffman [36], based on modifications from Hoffman [43] and the NRC (2001 [42]), suggested breeding and prepartum BW targets at 55% and 94% of the MBW, respectively. Van Amburgh et al. [44] concluded that optimization of the first lactation milk yield is feasible when the heifer BW post calving ranges between 82 and 85% of the MBW. More recently, the National Academies of Science, Engineering and Medicine (NASEM) suggested 55% MBW as the breeding BW goal and 82% MBW as the target BW for first calving, following the previous edition of the NRC (Table 1 [42,45]).

At first calving, increased BW is considered essential to avoid nutrient partitioning toward growth [44]. Moreover, lower conception and pregnancy rates are more commonly observed in lighter heifers [46,47], while heifers with a heavier BW at breeding (291–316 kg) tend to calve earlier compared with heifers with a lower BW at breeding (≤290 kg [48]). Handcock et al. [49] highlighted the importance of reaching higher BWs before breeding and demonstrated that an increased BW at 6, 12 and 15 months old increased heifer survival through the third lactation and likely resulted in earlier calving compared with lighter heifers. However, a slight decrease was observed in the reproductive performance and survival of heifers belonging to the upper BW range compared with the mid-range heifers [49]. Moreover, an increased BW at first calving was suggested, as a positive relationship between first lactation milk yield and BW at first calving was reported [39]. Handcock et al. [50] examined the effects of BW at calving to milk production from the first to third lactation periods. They found a positive curvilinear relationship between the milk yield and BW and reported that for a heifer calving at 21 months old, an increase in BW at first calving from 425 to 450 kg resulted in increases of 168 and 509 kg of energy-corrected milk in the first and third lactation periods, respectively [50]. Han et al. [51] examined the effect of a heifer’s BW at calving on the milk yield at first lactation and at 24 months after first calving, analyzing records from 1110 and 1229 cows from two different herds (Penn State University (PSU) herd and the University of Florida (UF) herd, respectively). The milk yield at the first lactation period and 24 months after first calving was analyzed by a linear model, taking into consideration the effects of BW quintiles at calving and MBW and BW changes occurring in the first month of milk production. The BW at calving ranged from 477 to 624 kg and from 473 to 604 kg, while the BW as a percentage of the MBW ranged from 68.9 to 87.9% and from 67.8 to 85.9% for the PSU and UF herds, respectively. The heaviest heifers produced more milk in the first lactation period (10,034 kg and 9683 kg for fifth and first quintiles, respectively), while no difference was observed in the milk yields 24 months after first calving. Additionally, the heaviest heifers lost more BW during the first month of milk production (3.65% and 3.99% BW change for heifers in the fourth and fifth quintiles, respectively) and had a higher risk of culling due to significant BW loss. Thus, in contrast with other research findings, they suggested heifers calving at 73–77% MBW (first and second quintile heifers) and concluded that any increase in BW after the third quintile of BW was not associated with the first lactation milk yield. More recently, Lauber and Fricke [52] demonstrated that primiparous cows that had attained 92% and 85% of MBW post calving, had higher mean milk production at 8 weeks of lactation compared with cows attaining 75% and 81% of the MBW post-calving (38.4 and 36.7 kg/d as well as 33.8 and 35.9 kg/d, respectively). However, similar to the study of Handcock et al. [49], heifers who attained 92% MBW post-calving calved 20 days later than leaner heifers (75% MBW), and 53.6% of the heavier heifers failed to conceive at first breeding, compared with 27.9% of the leaner heifers that failed to conceive at first breeding [52].

All things considered, determination of the MBW and the establishment of growth targets at benchmarks is important for allowing heifers to express their genetic potential. There are indications that by nearly reaching the MBW in the first lactation period, other parameters can be compromised, such as survival and conception rates. A moderate-to-high BW post calving at 85% of the MBW appears to benefit the first milk production levels without negative consequences. Taking into consideration that targets are breed- and herd-specific, the average AFC of the dairy herd should be evaluated. Then, efforts should be made to determine the average growth status of each age group and set specific growth targets (as a percentage of the MBW) for each group. Therefore, according to each growth stage, growth monitoring may enable achievement of the growth targets that have been set and eventually be useful for controlling the AFC.

## 4. Nutritional Management of Dairy Heifers

### 4.1. Physiological Basis and Nitrogen Metabolism

Increasing the NUE of growing heifers would not be feasible without understanding the mechanisms behind protein and carbohydrate metabolism and utilization. Replacement heifers have protein requirements for growth, maintenance and pregnancy, with the required amount of protein being dependent on the growth stage [45]. Dietary protein, and especially rumen-degradable protein (RDP), is used to provide true protein (TP) and non-protein N (NPN), which are converted into rumen amino acids (AAs) and NH_3_, and depending on carbohydrate availability, both are used for microbial protein synthesis [53]. Microbial protein in combination with rumen undegradable protein (RUP) provide AAs that are later absorbed in the small intestine, with microbial protein accounting for 50–80% of the total protein absorbed [54,55]. The AAs reaching the intestine comprise the metabolizable protein (MP) provided to the animal and are used for tissue deposition and growth [54].

When excessive rumen NH_3_ is accumulated in the rumen, the surplus is transported to the liver and converted to urea [53]. The amount of N consumed by the animal and not partitioned into body protein accretion is eventually excreted in feces and urine [56]. In contrast, when dietary protein is limited, the urea transported in the liver will be recycled back to the rumen through the rumen epithelium or salivary secretions to maintain a positive N balance [57,58]. In growing heifers, urea recycling appears to be driven by N intake, with heifers consuming less N and incorporating more N into microbial proteins [22]. Specifically, heifers fed diets containing CP levels of 9.1, 15.6 and 21.3% DM used 43.0, 25.0 and 6.0% N recycled for microbial protein synthesis, respectively [22]. Ruminants can survive solely on NPN sources [59]. However, growth cannot be exclusively supported by microbial protein synthesis [60], meaning that RUP is essential to meet the AA demands of a growing heifer [61]. Certainly, the first step is to maximize N use efficiency in the rumen, minimizing N losses in the form of NH_3_.

In this direction, synchronizing energy and protein supplied to rumen microorganisms has been suggested as a method to enhance microbial growth and, therefore, improve nutrient utilization [62,63]. Through carbohydrate fermentation, degradation to simple sugars (glucose, hexoses and pentoses) and, finally, glycolysis, adenosine triphosphate (ATP) is generated and used for microbial growth [63], while rumen microorganisms produce volatile fatty acids (VFAs) (mainly acetate, propionate and butyrate), which late become an energy source for the heifer. Furthermore, based on the Cornell Net Carbohydrate and Protein System (CNCPS), structural carbohydrate-fermenting bacteria require mainly NH_3_, and non-structural carbohydrate-fermenting bacteria require mainly AAs and less NH_3_ to meet growth demands [64], suggesting that protein degradation takes place first to release these AAs. However, ruminal degradation of different protein and carbohydrate fractions occurs at different rates depending on the source, with each fraction exhibiting different passage rates from the rumen as well [65]. For example, soluble proteins (SPs) and sugars are rapidly available in the rumen, while proteins bound to NDF (NDIPs) and NDF are slowly degraded in the rumen. Thus, carbohydrate and protein fractions supplied to the rumen determine the amount of energy and protein available for microbial protein synthesis. For maximizing microbial protein synthesis, it is important to meet microorganisms’ growth requirements and ensure the synchronous release of available energy and NH_3_ in the rumen.

Mechanistic models may be valuable tools for assisting with nutrient synchrony, especially because they contain functions to balance for microbial protein synthesis, considering both energy and protein availability (e.g., the rumen NH_3_ balance in CNCPS). These models are not dynamic, considering a static daily supply of nutrients in one meal [8,45]. However, heifers have demonstrated a feeding pattern with several meals taken within a day [66,67]. Perhaps this may explain why the CNCPS underestimates rumen microbial N outflow and overestimates rumen feed N outflow, though it still reaches an accurate rumen non-ammonia N outflow prediction [8]. Ongoing efforts to develop the CNCPS in a dynamic platform (version 7) may further improve its accuracy and precision and thus assist in formulating diets with optimal energy and protein synchrony.

In this direction, the accurate prediction of protein requirements and thus precise protein feeding is of high importance when it comes to minimizing N losses [15]. An important step was the shift between CP-based nutritional systems and digestible CP systems that take into consideration MP requirements, RDP requirements and intestinal AA absorption [54,56,68]. The recent recommended protein requirements based on the NASEM (2021 [45]) of 224 and 420 kg replacement heifers, gaining 0.84 kg/d and a MBW of 700 kg, are 610 and 772 g/d MP, respectively. An available nutritional model that balances rations based on MP is the mechanistic CNCPS. Van Amburgh et al. [69] evaluated the prediction accuracy of the CNCPS (version 3) and NRC (1989 [70]) for the nutrient requirements of growing heifers and demonstrated that the CNCPS was more accurate for estimating MP requirements (R^2^ = 0.76 compared with 0.68 for the CNCPS and NRC, respectively). Later, Fox et al. [40], based on the Beef NRC (1996 [71]), refined the heifer body composition and nutrient requirement equations, which were then adopted by the NRC (2001 [42]). Currently, the CNCPS for growth is based on the Beef NRC (1996 [71]), which is the same as the NRC (2001 [42]). However, the CNCPS is considered a diet formulation model with high sensitivity, especially in low-protein diets. In a dataset with a mean milk yield of 34.6 kg/d, when MP was the first limiting factor (relatively low supply of N), the updated version of CNCPS, version 6.5, predicted the MP-allowable milk and first limiting nutrient with errors of 1.1 kg and 1.6 kg of milk, respectively [8].

Overall, understanding N metabolism can define the extent to which manipulation of the diet composition can intervene with the aim to affect N utilization, while the use of mechanistic nutritional models proven sensitive to low-protein diets may be a valuable tool to further minimize N losses.

### 4.2. Effects of Crude Protein and Rumen Degradable Protein Level on Nitrogen Use Efficiency of Replacement Heifers

In the direction of decreasing N losses, an increase in replacement heifer NUE can be achieved through reducing the N intake without impacting growth. Taking into consideration the mechanisms of N utilization by the animal, there are certain dietary alterations that can be implemented to achieve this. Zanton and Heinrichs [72] observed that even though N retention and digestibility were not different between younger heifers and older heifers that were fed high- and low-energy density diets, the younger heifers retained higher levels of N when N retention was expressed as a proportion of consumed or digested N. They attributed the higher N utilization to better post-absorptive metabolism. However, it is well established that in the period from birth until weaning, there is higher body protein accretion than in other phases [44]. Therefore, the period from birth until weaning is expected to be the most efficient one. Chapman et al. [73] provided milk replacers containing two different levels of CP (20% and 26% DM), forming three treatments: (1) conventional, where claves were fed 446 g of DM per day of the 20% CP milk replacer; (2) moderate, where calves were fed 669 g of DM per day of the 26% CP milk replacer; and (3) aggressive, where calves were fed 892 g of DM per day of the 26% CP milk replacer. All groups were offered a starter containing 20.7% CP DM was offered ad libitum. The NUE ranged from 44.4 to 52.7%, with calves in the conventional treatment exhibiting higher efficiency. No difference was observed between the moderate and aggressive treatments (46.7 and 44.4%, for moderate and aggressive treatments, respectively), meaning that the increased CP supply reduced the NUE.

After weaning and until the first calving, it is expected that the NUE will be lower compared with the first stage of life, as N retention is reduced while the heifer reaches maturity [74]. Several studies have examined the appropriate supply level of CP, but the findings for N retention appear to vary (Table 2).

In an effort to provide an insight into the NUE of replacement heifers, we gathered 16 published studies related to N metabolism, excretion and retention, and a dataset was created. The dataset included studies that reported the BW, N intake, fecal N, urinary N and CP level. Then, the studies were categorized based on age, and the NUE was calculated as follows: NUE, % = (N intake − (fecal N + urinary N))/N intake × 100. The mean and standard deviation (SD) were calculated for each variable of interest. As is shown in Table 2, the phase before weaning exhibited the highest NUE. However, it is evident that there is high variation regarding NUE, both in the preweaning stage and in later growth stages, especially after 8 months of age. It is interesting that NUE in the 15–18-months-old group slightly increased arithmetically, while the CP levels of the studies examined were of approximately the same level, which can be considered a moderate CP level.

Marini and Van Amburgh [22] fed 265-kg heifers diets containing CP levels of 9.0, 11.8, 15.6, 18.6 and 21.2% DM at a forage-to-concentrate ratio (F:C) of 30:70%. The authors found that the N balance, N recycled and microbial protein levels were better for heifers fed diets at 9.0 and 11.8% DM, leading to NUE of 22.4% in both cases compared with 20.0, 18.1 and 15.9% for 15.6, 18.6 and 21.2% CP levels of DM, respectively. Hoffman et al. [26] offered heifers of 410-kg BW diets containing levels of CP at 8.0, 11.0, 13.0 and 15.0% DM. Even though the ADG did not differ among treatments, the maximum N retention was observed at a CP level of 13.0% DM, resulting in the highest NUE at 13.7%. Johansen et al. [78] fed 340- and 435-kg heifers (12 and 16 months, respectively) three levels of CP at 12.2, 14.6 and 17.0% DM and 9.8, 12.2 and 14.6% DM, respectively. Based on their data, we calculated that the highest N retention for 12-month-old heifers was found in diets containing a CP level of 12.2% DM, with NUE at 15.6%, while at the same CP level, the NUE of the 16-month-old heifers was 8.5%. Adachi et al. [84] fed heifers at 9 weeks prepartum diets containing a low and a high level of CP at 11.5 and 14.1% DM, respectively. It was calculated that the NUE was 22.1 and 36.9% for the low and high groups, respectively. In contrast, Johansen et al. [78] fed 20-month-old heifers at 161 d of gestation diets with 9.8, 12.2 and 14.6% DM of CP, resulting in NUE levels of 14.7, 11.5 and 7.04%, respectively.

Within our dataset, there were studies included that used limiting feeding of heifers as a strategy to reduce the DM excreted and therefore minimize the amount of nutrient losses. Zanton and Heinrichs [81] implemented limited feeding for 12-month-old heifers at two forage levels (LF = 25% forage; HF = 75% forage) and tested four levels of N intake: (1) low CP at 7.9 and 7.5% DM for LF and HF, respectively, (2) medium-low CP at 13.7 and 12.6% DM for LF and HF, respectively, (3) medium-high CP at 19.5 and 17.8% DM for LF and HF, respectively, and (d) high CP at 25.4 and 22.9% DM for LF and HF, respectively. They observed that fecal and urinary N excretion increased with increased levels of N intake, with N retention increasing linearly as well. Indeed, based on their data, it was calculated that the NUE was 18.0 and 15.8% at low N intake for LF and HF diets, respectively, 23.0 and 25.7% at medium-low N intake for the LF and HF diets, respectively, 26.0 and 24.9% at medium-high N intake for LF and HF diets, respectively, and 33.1 and 24.7% at high N intake for LF and HF diets, respectively. However, N retention was not different between forage levels. In the above study, the forage and N levels were primarily examined, without investigating the effects of limited feeding on N utilization. Hoffman et al. [83] limited the feeding of pregnant heifers 17.5 months in age with diets containing CP at 11.3%, 12.7% and 14.2% DM and the DMI set at 100, 90 and 80% of the control diet, respectively. They found that heifers consuming 80 and 90% of the control diet had lower N retention levels. Moreover, no difference was observed in N utilization, and even though reduced DM excretion was observed in the limited-feed heifers, the N excretion and NUE did not improve, resulting in NUE levels of 23.2, 21.8 and 19.4% for heifers in the 100, 90 and 80% control DMI groups, respectively.

**Figure 1:**
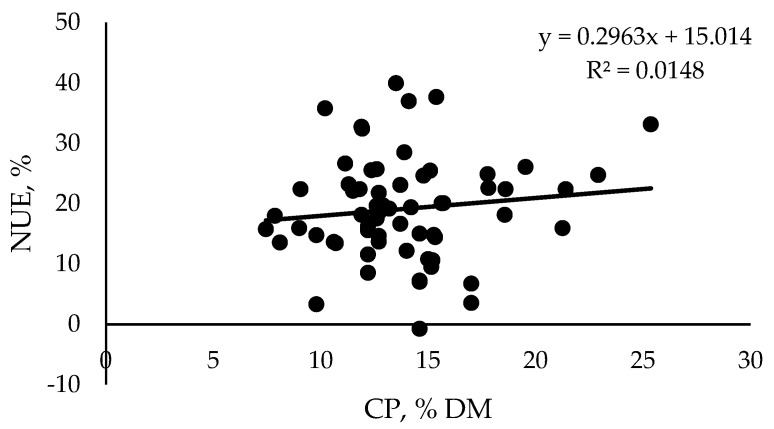
Nitrogen use efficiency in relation to dietary crude protein levels of 5–24 month old dairy replacement heifers.

Ιt is evident that during the period from weaning to calving, there is high variation regarding the NUE and CP levels. Specifically, the NUE results were plotted against the CP levels using study results regarding replacement heifers 5–24 months old, with a BW range from 166 to 657 kg. As a result, a weak relationship between the NUE and CP levels was observed (R^2^ = 0.0148; Figure 1). It is obvious that diets with higher contents of CP did not necessarily bring any further improvement in N utilization, meaning that a moderate CP level might reduce N excretion and improve NUE. In addition, this highlights the nutritional importance of MP fed to animals, since it is well established that the CP level alone is not an accurate measurement of the N supply.

Another strategy to reduce NH_3_ losses is the alteration of protein degradability in the rumen [86,87]. Once the microbial requirements for growth have been met from RDP sources, RUP may further improve N retention [88]. Silva et al. [79] examined the effects of RUP levels on N utilization of 276-kg replacement heifers. The diets contained levels of RUP at 38.9, 44.1, 48.9 and 54.0% CP, while the CP levels were similar among the treatments at 15.1, 15.2, 15.3 and 15.3% DM, respectively. The nitrogen balance was found to be lower for the highest RUP levels in diets with 48.9 and 54.0% CP, with N intake and N fecal excretion not being affected by the RUP level. A tendency for reduced urinary N and increased N retention was observed with increased levels of RUP, with NUE increasing from 9.5, 10.6, 14.8 and 14.4% for the higher levels of RUP at 38.9, 44.1, 48.9 and 54.0% CP, respectively. They concluded that the higher levels of RUP were similar and proposed that 48.9% RUP of CP can be utilized in the diets of growing heifers that need to maintain high growth rates. In contrast, under tropical conditions, Corea et al. [77] tested two levels of RUP (26 and 36% CP) with the same CP level (12.6–12.9% DM) and two different forage sources (cowpea hay and pangola grass hay). They observed no effect from different RUP levels on N excretion and retention. Based on their data, we calculated NUE levels of 17.5 and 19.6% for the diets containing RUP at 26 and 36% CP under pangola grass hay treatment, respectively, and 18.5 and 19.7% for the diets containing RUP at 26 and 36% CP under cowpea hay treatment, respectively.

As a strategy to increase nutrient utilization, Zanton et al. [82] proposed an increase in SP content when replacement heifers were fed diets relatively high in forage and thus high in the NDF content. They examined the effects on the SP and RUP levels of 17-month-old heifers fed high-forage diets in a 2 × 2 factorial experimental design (low-SP treatment: low RUP (RUP 38.1% CP, SP 27.3% CP) and high RUP (RUP 43.9% CP, SP 26.6% CP); high-SP treatment: low RUP (RUP 38.7% CP, SP 35.6% CP) and high RUP (RUP 46.7% CP, SP 37.7% CP)) and observed no effect on N utilization. Based on their data, the NUE was calculated to be 13.6 and 26.6% for the low-SP treatment with low RUP and high RUP contents, respectively. For the high-SP treatment with low RUP and high RUP contents, the NUE was calculated to be 32.4 and 25.5%, respectively. Similarly, Lascano et al. [61] investigated the effect of high RDP levels on N utilization in diets with low or high forage contents in limited-feed heifers. Heifers 15.5 months in age were assigned to a 3 × 3 Latin square design and two dietary treatments with different F:C ratios, namely with low forage (LF) and high forage (HF) at F:C ratios of 45:55 and 90:10, respectively, with three different forage proportions of (1) 33% wheat straw and grass hay and 67% corn silage (33% treatment), (2) 50% wheat straw and grass hay and 50% corn silage (50% treatment) and (3) 67% wheat straw and grass hay and 33% corn silage (67% treatment). The CP and RDP levels of the diets ranged from 10.5 to 11.4% DM and from 64.0 to 66.3% CP for the HF heifers and from 12.2 to 12.7% DM and from 63.8 to 64.0% CP for the LF heifers, respectively. No difference was observed in urinary N excretion, but N retention was higher for all LF heifers, with heifers under the 33% treatment exhibiting the highest retention at 69.4 g/d compared with 53.3 g/d for the HF heifers under the 33% treatment. Based on these data, it was calculated that the NUE was highest for the group under the 33% treatment of LF heifers at 37.6%, while the lowest NUE had 67% inclusion of HF heifers at 14.6%. The authors concluded that heifers on the LF diets benefited the most from high RDP levels, while the HF diets presented poor N and nutrient utilization, highlighting the importance of higher RUP levels with higher levels of forage inclusion under limit feeding conditions.

Nevertheless, no difference in microbial crude protein was observed between the two F:C ratios. In fact, authors reported a linear decrease in microbial crude protein with the increase in fiber content, especially in LF treatment. Usually, a decrease in microbial protein synthesis is observed due to a reduction in rumen N and the energy supply or an absence of protein and carbohydrate synchronization [89]. Indeed, in the 67% HF treatment diet, the starch level was at 15.1% DM, derived mainly from corn silage, while in the 67% LF diet, the starch level was at 29.8% DM, supplied mainly by cracked corn. The NDF levels were higher for the 67% HF diet compared with the 67% LF diet (56.8% and 36.0% DM for HF and LF, respectively). In both cases, high levels of RDP and SP were supplied. Regarding HF diets, it appears that microbial growth was limited by the asynchronous release of protein and energy, meaning the lower concentration of highly fermentable carbohydrates did not allow the SP to be efficiently utilized. In contrast, as Lascano et al. [61] suggested, in low-forage diets containing less than 20% forage, the increase in structural carbohydrates may have further improved N utilization and thus microbial growth, while above 45% forage, additional fiber negatively impacted microbial protein synthesis. When we inputted the diets into the CNCPS (version 6.5), it was found that in both diets, metabolizable energy was the limiting factor. The rumen NH_3_ balance was adequate in both cases, while the MP in the 67% LF diet vastly exceeded the requirements. Therefore, it is evident that under in vivo conditions, not only should the forage source, fiber level, and energy availability be considered but also specific fractions and their energy and N degradation patterns. This highlights the importance of using mechanistic models to predict nutrient supplementation in order to maximize microbial protein synthesis efficiently with the lowest economic and environmental cost. In any case, this can happen under a low- or high-forage diet, including, however, the proper feeds in terms of degradability. Whether following a low- or high-forage diet, limited or ad libitum feeding under commercial conditions depends on management’s decision making. However, beyond nutrient synchrony, other parameters should be taken into consideration (e.g., energy requirements, adipose tissue deposition and welfare).

Overall, the effect of different RDP and RUP ratios on NUE is not conclusive. In many cases, the lack of effect may be attributed to age and BW differences, actual degradability of feeds and following different feeding strategies. It appears that RDP and RUP alone cannot be used to provide nutritional recommendations. In contrast, mechanistic models, such as the CNCPS and NASEM, differentiate protein fractions based on degradability and solubility and consider microbial N requirements. They provide better insight into the N balance in the rumen (e.g., Rumen NH_3_ balance in the CNCPS), which can be used instead. Practically, once microbial protein synthesis is maximized, based on the available RDP and energy in the rumen, RUP may be used to maximize the MP supply [90]. In this direction, mechanistic nutritional models are useful tools to adequately meet heifer requirements and avoid N losses.

## 5. Conclusions

Even though N is essential for sustaining life and productivity, its reactive forms are transferred through atmospheric, aquatic and terrestrial ecosystems, negatively impacting environmental balance and creating various pollution phenomena. Increasing the NUE of dairy herds should take into consideration replacement heifers, since they comprise a vital part. The control of AFC and determination of growth targets are the first steps toward minimizing N losses from the replacement herd. Moreover, proper diet formulation that meets an animal’s requirements at each growing stage is essential for improving farm NUE.

## Figures and Tables

**Table 1:** Coefficients of growth targets expressed as percentage of mature body weight (MBW) for dairy replacement heifers based on different suggestions.

	MBW (%)				
Stage	Hoffman [36]	Van Amburgh et al. [39]	Fox et al. [40]	NRC, 2001 [42]	NASEM, 2021 [45]
Breeding	55	55–60	-	55	55
Prepartum	94	-	-	-	91
1st Calving	-	82	85	82	82
2nd Calving	-	-	92	92	92
3rd Calving	-	-	96	100	100

**Table 2:** Average nitrogen use efficiency (NUE) based on age and crude protein (CP) levels of diets fed to dairy replacement heifers of different body weights (BWs).

Age (Months)	BW (kg)	SD	CP (% DM)	SD	NUE (%)	SD	Reference
Preweaning	-	-	22.9	5.1	50.7	9.3	[73,75,76]
5–7	221	52	14.2	3.8	19.4	2.1	[25,77]
8–9	261	15	14.4	3.2	19.6	9.7	[22,78,79,80]
10–12	372	16	14.5	5.3	17.9	8.1	[26,78,81]
15–18	466	17	12.9	1.7	20.2	10.3	[61,78,82,83]
20–24	576	69	13.5	2.4	17.8	9.3	[78,84,85]

## Data Availability

Data sharing is not applicable. No new data were created or analyzed in this study.
